# Extracellular vesicles: An emerging platform in gram-positive bacteria

**DOI:** 10.15698/mic2020.12.737

**Published:** 2020-10-05

**Authors:** Swagata Bose, Shifu Aggarwal, Durg Vijai Singh, Narottam Acharya

**Affiliations:** 1 Department of Infectious Disease Biology, Institute of Life Sciences, Bhubaneswar-751023, India.; 2 Department of Biotechnology, School of Earth, Biological and Environmental Sciences, Central University of South Bihar, Gaya-824236, India.

**Keywords:** virulence, antibiotic resistance, peptidoglycan, HGT, biofilm, pathogenesis, quorum sensing, immune response, extracellular DNA, vaccine

## Abstract

Extracellular vesicles (EV), also known as membrane vesicles, are produced as an end product of secretion by both pathogenic and non-pathogenic bacteria. Several reports suggest that archaea, gram-negative bacteria, and eukaryotic cells secrete membrane vesicles as a means for cell-free intercellular communication. EVs influence intercellular communication by transferring a myriad of biomolecules including genetic information. Also, EVs have been implicated in many phenomena such as stress response, intercellular competition, lateral gene transfer, and pathogenicity. However, the cellular process of secreting EVs in gram-positive bacteria is less studied. A notion with the thick cell-walled microbes such as gram-positive bacteria is that the EV release is impossible among them. The role of gram-positive EVs in health and diseases is being studied gradually. Being nano-sized, the EVs from gram-positive bacteria carry a diversity of cargo compounds that have a role in bacterial competition, survival, invasion, host immune evasion, and infection. In this review, we summarise the current understanding of the EVs produced by gram-positive bacteria. Also, we discuss the functional aspects of these components while comparing them with gram-negative bacteria.

## INTRODUCTION

Bacterial pathogens quickly respond to changes in the environment to survive and propagate. Pathogenicity of a bacterium is a measure of virulence, which is manifested by the secretion of bacterial virulence factors via membrane blebs for invasion. The secretory vehicles called extracellular vesicles (EVs) are nanoparticles produced by most of the bacteria which have diverse biological functions and broad applications in immunology and biotechnology [1]. The formation of vesicles appears to be a conserved process in both pathogenic and non-pathogenic bacteria. During the past few years, EVs have gained attention in all domains of life. EV release is now considered as a primordial feature of all living cells [2, 3]. An EV is defined as a spherical, membranous vesicle generated from a microbial cell surface with size ranging from 20 nm to 500 nm in diameter. Not only do they differ in size, but they also vary in morphology, composition, and biogenesis. EVs have been coined different names in different organisms such as OMVs (outer membrane vesicles) in gram-negative bacteria and EVs or MVs (extracellular vesicles or membrane vesicles) in gram-positive bacteria. However, to maintain uniformity; the International Society for Extracellular Vesicles (ISEV) recommends EV as a collective term for “particles naturally released from the cell that is delimited by a lipid bilayer and cannot replicate” [4]. EVs contain various macromolecules such as proteins, nucleic acids, phospholipids, adhesins, and lipopolysaccharides required for virulence, sensing nutrition, and cell-cell communication. Several reports suggest that excess of EVs can be produced due to an abnormality in cell envelope in response to certain stress.

**Figure 1:**
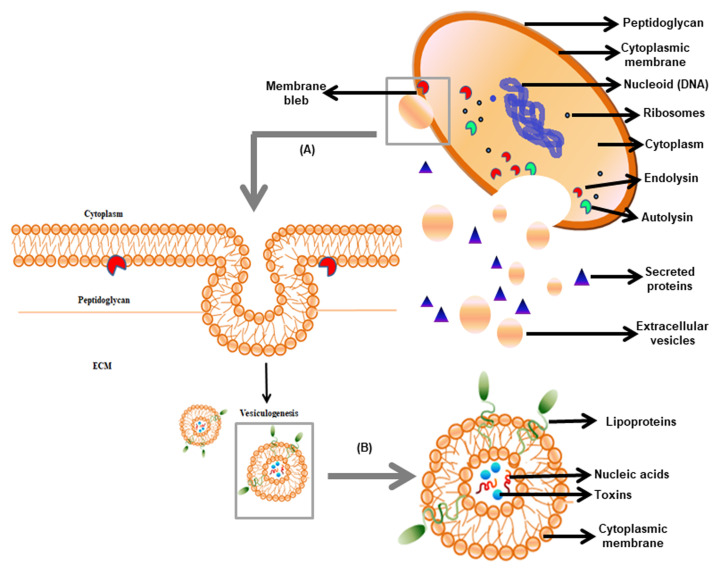
FIGURE 1: Origin and composition of gram-positive EVs. **(A)** The release of EVs involves diversified events depending on the cell lytic enzyme viz. PGN degradation followed by cytoplasmic membrane bleb protrusion via endolysins and PGN remodelling via hydrolyzing enzyme autolysins. **(B)** The internal structure of EVs comprising of nucleic acids, virulence factors, and intracellular proteins.

In the 1960s, bacterial EVs were first reported in *Escherichia coli,* but their existence in gram-positive bacteria gained attention recently [5, 6]. In 1990, Dorward and Garon [6] provided the first evidence of vesiculation in gram-positive bacteria (**Figure 1**). The release of spherical particles occurs by budding in the surrounding environment from the cells. It has been observed in several bacterial species belonging to gram-positive phyla Firmicutes and Actinobacteria [7]. EVs of gram-positive bacteria are of nanometer-size ranging from 10-500 nm and comprised of a simple architecture containing cell membrane and cytoplasm. Membrane and lumen of the vesicles of the bacteria are derived from the cytoplasmic membrane and the cytoplasm, respectively [8]. Similar to gram-negative bacteria, the EVs in gram-positive bacteria consist of proteins, nucleic acids, and toxins. Besides, gram-positive bacterial EVs do not contain periplasmic components, which is present in gram-negative bacteria [9, 5]. EVs are involved in cell-to-cell communication, sensing nutrients, elimination of competitors, and detoxification of environmental stress. Protein secretion via EVs to extracellular milieu independent of soluble secreted proteins has gained attention as it contributes to pathogenesis. Limited studies on EV release have been shown in few gram-positive bacteria that are relevant to human diseases e.g. *Staphylococcus aureus, Listeria monocytogenes, Clostridium perfringens, Streptococcus pneumoniae, Mycobacterium ulcerans,* and *Bacillus anthracis.* Lee *et al.* (2009) reported for the first time that *S. aureus* naturally produces EVs [10]. Hong *et al.* [11] revealed the presence of pathogenic molecules in *S. aureus* EVs that may be involved in the pathogenesis of atopic dermatitis. Also, *S. aureus* EVs were shown to enhance the development of airway hypersensitivity to inhaled allergens [12]. The isolation of EVs from the culture supernatant of *B. anthracis* is considered as a powerful biological weapon. These findings clearly showed that vesicles in gram-positive bacteria carried lethal factors and deliver toxins to the host cells [13], thereby showed significance in pathogenesis. In this review, we discuss the roles of EVs in physiology, host-microbial interaction, and their implications in the development of vaccines and antibiotics effective against the pathogenic strains of gram-positive bacteria.

## STRUCTURE AND COMPOSITION OF EVs IN DIFFERENT BACTERIA

Microbial EVs showed certain differences in their morphology and composition. Whereas EVs adhere and fuse rapidly with the outer membrane in gram-negative bacteria, they just attach to the membrane surface in gram-positive bacteria [14]. Shockman and Barrett [15] showed that the architecture of the cell wall in gram-positive bacteria is analogous to that of mycobacteria and fungi, suggesting a similar process of EV biogenesis. EVs released from gram-negative bacteria are spherical and bilayered with size ranging from 10 nm to 300 nm in diameter [16, 15]. However, the gram-positive bacteria EVs are of ~20-150 nm diameters, derived directly from the cytoplasmic membrane [16]. *Streptomyces coelicolor* EVs are found to be unilamellar with a bilayer membrane ranging from 80–400 nm in diameter with a width of 150-300 nm [17]. EVs of 20–100 nm diameter in the culture filtrate of *S. aureus* were detected by using a transmission electron microscope (TEM) [10]. Similarly, *Bacillus* sp. and other gram-positive bacteria were found to shed EVs in the size range of 50 - 150 nm diameters [10, 13]. On the basis of structural and molecular studies, the composition of gram-negative and gram-positive vesicles was analyzed. The gram-negative bacterial EVs contained peptidoglycan, virulence factors, inner membrane, cytoplasmic proteins, DNA, and RNA. On the other hand, the EVs of gram-positive bacteria contained fatty acids, phospholipids, cytoplasmic proteins, membrane-associated virulence proteins, lipoteichoic acid, peptidoglycan, DNA, and sRNAs [16]. The proteomic analysis showed that the vesicles contain proteins and chaperones required for cell wall architecture and carbohydrate metabolism [18]. However, the lipidomic analysis showed that EVs of *B. anthracis* and *S. pneumoniae* contain fatty acids like myristic acid and palmitic acid [19]. Similarly, analysis of *Staphylococcus* EVs showed the presence of fatty acids [20, 21]. The predominant fatty acids found in EVs are palmitic, octadecenoic, stearic, and eicosenoic acids. A summary of the morphology and composition of EVs of gram-negative and gram-positive bacteria is given in **Table 1**.

**TABLE 1.** Comparison between gram-negative and gram-positive extracellular vesicles.

**Sl. No.**	**Features**	**Gram-negative EVs**	**Gram-positive EVs**	**Ref**
1	Origin	Outer membrane	Cytoplasmic membrane	[16]
2	Size	10 nm-300 nm	20 nm-400 nm	[16]
3	Components	Outer membrane proteins, periplasmic proteins, virulence factors, cytoplasmic proteins, inner membrane proteins, lipopolysaccharides, phospholipids, and peptidoglycan (10%-20%)	Cytoplasmic proteins, membrane-associated proteins, lipoteichoic acid (LTA), peptidoglycan (>50%)	[16]
4	Genetic components	sRNA, mRNA, miRNA, luminal and surface associated DNA	sRNA, extracellular and chromosomal DNA	[16]
5	Proteins	Outer membrane: OmpA, OmpC, OmpF, lipoprotein (Lpp), periplasmic: Alkaline phosphatase and AcrA	Single lipid membrane proteins: penicillin-binding, immunoglobulin G-binding (protein A), staphopain A, α-haemolysins, heat-shock protein	[9, 50]
6	Lipids	Glycerophopholipids, phosphatidylethanolamine, phophotidylglycerol and cardiolipin	Phosphatidylglycerol, myristic and palmitic acids	[9, 19]
7	Coagulation	E-selectin, P-selectin, thrombomodulin	Fibronectin binding protein, *staphylocoagulase precursor*, Vonwillebrand factor binding protein	[9]
8	Antibiotic resistance	β-lactamase, enzyme L5, multidrug efflux protein (*Mtr, Mex, TolC*)	β-lactamase, Penicillin-binding proteins: PBP1, PBP2, PBP2a, PBP3 and PBP4	[9]
9	Virulence factor delivery	Enzymes: phospholipase C, esterase lipase, alkaline phosphatase, serine protease Toxins: adenylatecyclase, cholera, cytolethal distending, PagJ, PagK1, VacA	InIB, LLO, IgG binding protein SbI, protective antigen, lethal factor, edema toxin, anthrolysin	[9]
10	Bacterial survival	Hemin-binding protein, TonB-dependent receptors	β-lactamase protein	[9, 39]
11	Bacteria adhesion & invasion	Adhesin/invasin, OmpA	Plasma binding proteins, staphopain A	[9]
12	Immune evasion	Cytotoxic necrotizing factor 1, UspA1/A2	Coagulation factors, antibody degradation and sequestering factors, complement inhibition factors	[7, 9]
13	Host-cell modulation	Cytolysin A, VacA toxin, CNF1, heat-liable enterotoxin, shigatoxin, Cif, flagellin, α-haemolysin	Proteolysin, β2 toxin	[9]
14	Killing competing bacteria	Endopeptidase L5, murein hydrolase (Mtl, Slt), peptidoglycan hydrolase	N-aetylmuramoyl-L-alanine amindase	[9]
15	Biogenesis	a. Loss or relocation of covalent linkages between the OM and the underlying peptidoglycan layer b. Accumulation of peptidoglycan fragments in the outer leaflet of the OM c. Misfolded proteins in periplasmic space exerting turgor pressure on OM d. Enrichment of species-specific membrane curvature-inducing molecules	Action of cell wall-degrading enzymes; endolysin, autolysin	[7]

## EVs BIOGENENSIS AND PEPTIDOGLYCAN DEGRADING ENZYMES

In gram-negative bacteria, EVs are easily pinched-off from the outer membrane, due to the presence of a thin outer cell wall. In contrast, EV biogenesis in gram-positive bacteria is a complex process because of the presence of a thick peptidoglycan barrier. In almost all organisms, the outer layer provides a protective barrier against different stress conditions. Similarly, gram-positive bacteria possess a thick cell wall ranging between 20–40 nm that helps to withstand the extremities like osmotic pressure, DNA-damaging agents, exposure to antibiotics [22]. Kim *et al.* [9] reported that peptidoglycan (PGN) is the major component of the cell wall in addition to polysaccharides, proteins, and polymers. The thick PGN layer with a pore size of approximately 2 nm may prevent the release of EVs with a diameter of 20-400 nm [23]. Thus, the intriguing question that remains is “how EVs traverse the thick cell wall?” In *S. aureus,* it was demonstrated that the action of certain PGN degrading enzymes could remodel the cell wall in a way that may facilitate EVs to transit across the cell wall [10]. In *Bacillus subtilis*, a phage-encoded endolysin, which promotes the pore formation in peptidoglycan layers, facilitates the release of the EVs [24]. In this event, endolysins first weaken the PGN causing *B. subtilis* cells to protrude their cytoplasmic membrane, thus forming membrane blebs, and finally releasing it as EVs. Interestingly, the vesicles from *S. pneumoniae* were enriched in putative phage-associated endolysin [25]. A predatory role of *S. aureus* EVs has been proposed in which vesicle-associated N-acetylmuramoyl-L-alanine amidase can kill other competing bacteria by PGN degradation and cell lysis, similar to that observed with EVs of *Pseudomonas*

*aeruginosa* [14]. In community-associated methicillin-resistant *Staphylococcus aureus* (CA-MRSA) EVs, the cytoplasmic membrane is disrupted through phenol-soluble modulins (PSMs) [10]. In another study, it was shown that *S. aureus* uses PGN hydrolase activity of two autolysins Atl and Sle1 during cell division resulting in the separation of daughter cells [26, 27]. However, the role of autolysins in modulating EV release from the cell wall and its regulation is yet to be determined. In mycobacteria, the cell wall is made up of mycolyl-arabinogalactan-peptidoglycan (mAGP) complex. The genesis of EV in mycobacteria is thought to be due to the action of remodeling enzymes or proteins similar to that obtained in EVs biogenesis of *S. aureus* [28, 18]. All this information ascertains that the disruption of an extensive cross-link of PGN is possible only through the presence of degrading enzymes and surfactant proteins to facilitate EVs to escape from the thick cell wall of gram-positive bacteria (**Figure 1**).

## ROLE OF EVs IN ANTIBIOTIC RESISTANCE

Bacteria are usually sensitive to antibiotics but carry antibiotic resistance genes present on either chromosome or extra-chromosome that may lead to the development of antibiotic resistance [29]. As part of the adaptive response, pathogenic bacteria show a heavy yield of EVs compared to non-pathogenic bacteria [30, 31]. Factors like antibiotics, reactive oxygen species (ROS), lipopolysaccharide, serotype-switching influenced EV production in bacteria [32, 33]. EVs from *B. subtilis* is enriched in Sun1 protein (also known as *YolF*), which confers immunity to sublancin antibiotic [34, 35]. Moreover, the large quantity of vesicles from *B. subtilis* was found to be linked with the expression of the lipopeptide antibiotic surfactin. Surfactin destabilizes vesicles leading to the lower amount of recoverable vesicles in the pellet fraction [34].

DNA-damaging agents, UV exposure, and antibiotics induced lytic gene expression stimulate SOS response to trigger vesicle formation in lysogenic strains of *S. aureus* [36]. Likewise, genotoxic stress could promote bacterial lysis after DNA replication, phage assembly, and DNA packaging to release EVs and new phage proteins [24]. β-lactam antibiotics also weaken the PGN layer causing the cytoplasmic membrane protuberance to release it as EVs [25, 37, 38]. Proteomic analysis showed that *S. aureus* ATCC 14458 derived EVs contain *BlaZ*, a β-lactamase required to degrade β-lactams [10]. Liu *et al.* [7] suggested that resistant EVs confer protection to the susceptible bacteria by degrading ampicillin in the environment. Thus, it is clear that EVs play an important role in the establishment of antibiotic-resistant subpopulations either by the transfer of antimicrobial-resistant factors or by horizontal gene transfer (HGT). Andreoni *et al.* [36] reported that β-lactam antibiotics like flucloxacillin and ceftaroline weaken the PGN layer, thus increasing vesicle formation in a prophage-independent manner. Also, purified EVs from *S. aureus* protect bacteria from daptomycin, a membrane-targeting antibiotic. Lee *et al.* [39] reported that vesiculation could occur via both a phage-dependent and phage-independent manner in lysogenic strains of *S. aureus,* based on the nature of the antibiotics [39]. Actinorhodin, an antibiotic, was identified in EV-containing exudate of *S. coelicolor* [13]. It was suggested that vesicles can serve as decoys for phages and membrane targeting antibiotics and contribute to the survival of the bacterium [38]. In *Mycobacterium*, Cheng and Schorey [40] proposed that EVs released from *Mtb*-infected macrophages in combination with antibiotics can be used to treat drug-resistant tuberculosis (TB) [40]. In fact, EVs synergize with TB antibiotics to promote bacterial clearance and reduce lung pathology. Also, EVs increased the efficacy of the antibiotic moxifloxacin in mice infected with *Mycobacterium tuberculosis*. These observations thus suggest that EVs can provide an immunotherapeutic approach to treat drug-resistant *M. tuberculosis* [41].

## ROLE OF EVs IN VIRULENCE

The role of gram-positive EVs in infection and bacterial pathogenesis is now an emerging area of research [42, 43]. Since the virulence factors are the principal constituent of EVs, the focus lies on the role of EVs during infection [37]. It has been reported that in several pathogenic bacteria, the vesicles signify vesicular transport as a mechanism for concerted delivery to host cells and tissues [44]. In *Cryptococcus neoformans* and *B. anthracis*, vesicle content has been associated with virulence. While Cryptococcal vesicles that contain glucuronoxylomannan (GXM), is a potent immunomodulatory molecule, *B. anthracis* EVs containing several toxin components play a role in cytotoxicity [45, 13]. Moreover, *B. anthracis* vesicles can deliver their cargo directly to the macrophages either by phagocytosis or by fusion with the plasma membrane [44]. In *B. anthracis*, the components of anthrax toxin lethal factor (LF), edema factor (EF), and protective antigen (PA) were found in the EV pellet but not in the supernatant, suggesting encapsulation of toxins during EV packaging [13, 46].

Virulence factors promoting invasion and dissemination throughout the tissues have been identified in EVs. Collagenase and hyaluronate lyase are involved in promoting invasion, and extracellular matrix (ECM) but serine proteases, like exfoliative toxins, facilitate the disruption of the physical barriers [47, 48, 49]. Lee *et al.* [10] have demonstrated the presence of virulence-associated proteins in *S. aureus* EVs. *S. aureus* EVs delivered virulence factors to host cells in a way similar to that found in gram-negative bacteria [42]. In *S. aureus*, EVs carried virulence factors, super-antigens, and immunoglobulin-G binding proteins. Two major factors, namely Staphopain A and α-hemolysins, can induce apoptosis of the host cell by forming pores and causing cellular invasion [50–54]. EVs from *L. monocytogenes* include virulence factors listeriolysin O (LLO) and internalin B (InlB) which are necessary for cellular invasion and escape from host vacuoles [55, 56]. Moreover, pneumolysin present in the EVs of *S. pneumoniae* which is a pore-forming cytolysin is important for pathogenesis [57, 58]. In *Mycobacterium*, EVs are mainly composed of cytotoxins and other virulent factors. They infect the host cells by releasing their EV content [59]. In a study, Marsollier *et al.* [60] demonstrated that *Mycobacterium ulcerans* EVs containing cytotoxin mycolactone showed potent pathogenicity in the host when compared to its isolated form. Other workers also reported that the salicylate-derived mycobactin siderophores of *M. tuberculosis* are essential for bacterial growth in macrophages [61, 62].

## EVs AND HOST INTERACTION

EVs transport various immunomodulatory molecules depending on the pathogen's environmental conditions. Studies showed that EVs play a critical role in niche colonization, adhesion, the transmission of virulence factors, mitigating host immune response, and cytotoxicity. EVs of *B. anthracis* contain anthrolysin and other related active toxins that induce cytotoxicity in host cells [13]. In *S. aureus*, the delivery of virulent factors from intact EVs causes cytotoxicity in host cells in a dose-dependent manner [63]. Since the treatment of host cells with a cholesterol-destroying agent MβCD prevents the entry of EVs into the host cytosol, it was suggested that gram-positive bacteria follow a cholesterol-rich membranous micro-domain delivery mechanism while interacting with the host cell. Similarly, the PSMs are one of the major contributors in *S. aureus* pathogenesis and disease progression. PSM possessed surfactant-like property having a cytotoxic effect on leukocytes, epithelial, and endothelial cells of the host [64]. In another study, PSMα peptide showed its potential role in the formation of EVs from *S. aureus* [17]. These surfactant proteins deform the host lipid layer and trigger bacterial membrane curvature resulting in the production of excess EVs. Recently Wang *et al.* [65] found that EV-mediated *Staphylococcal* lipoproteins not only activate NLRP3 inflammasome but also alter the IL-1β, IL-18, and caspase-1 activity of the host. In Mycobacteria, EVs employ various techniques to disrupt host immune response [66]. Mycobacterial EVs directly interact with TLR2-signaling and contribute to infection [67]. Interestingly, the TLR2 association with Mtb ligands creates an ambiguity between the entities, as their interaction promotes bacterial clearance as well as an escape strategy for bacteria from the host immune response [68]. Lipoarabinomannan (LAM) in EVs was reported to inhibit CD4+ T-cells function and CD4+ T-cells are required for Mtb clearance [69].

## ROLE OF EVs IN HGT

In bacteria, in addition to transformation, transduction, and conjugation, the fusion of EVs carrying DNA between the cells is a mechanism for HGT [70, 71]. HGT plays a primary role in the evolution of many organisms through the movement of genetic materials, the spread of antibiotic resistance in bacteria, virulence factors, and pathogenicity. Various reports suggest that there is intra- and inter-species vesicle-mediated gene transfer from *Acinetobacter baumannii, Acinetobacter baylyi,* and *Pseudomonas aeruginosa* to *E. coli* cells [72]. The potential of EVs for inter-species gene transfer relies on the capacity of recipient species to take-up and maintain horizontally acquired DNA. Short linear chromosomal pieces and repetitive DNA sequences are further processed and packaged into EVs for export outside the cell [73]. MVs mediated sub-cellular lateral gene transfer (LGT) is responsible for encoding various metabolic enzymes in *Ruminococcus* species. For example, *Ruminococcus alba* vesicles play a role in the horizontal transfer of cellulolytic genes to degrade crystalline cellulose. In *S. aureus*, transduction is a vital gene transfer process, where mobile genetic elements (MGE) encodes for a varied range of proteins representing diverse virulence factors for antibiotic resistance and host adaptation. Through HGT, MGEs bring adjustment in the genetic level between different isolates of the same lineage at high frequency. Presumably, *S. aureus* relies on MGE for its selective advancements [74, 75]. Although several reports focused on MGE-mediated DNA transfer, the correlation between MGE in the context of EV remains elusive. In *Bacillus* species, about 112 transposase genes play an evolutionary role in HGT. Ten prophages are involved in gene transfer mechanisms leading to bacterial pathogenicity [76]. The proteome analysis showed phage proteins packaged into EVs as HGT mediators in *B. subtilis* [24]. It has been observed that such EVs contribute to phage integration into the host chromosome and eventually results in spreading and invasion [7]. Few reports suggest that the acquisition of sensitivity (ASEN) drives the exchange of genetic material between resistant and sensitive cells through phage-mediated EVs. The phage-resistant cells acquire sensitivity via phage receptors produced by susceptible cells [77]. A novel gene exchange mechanism was found in *Mycobacterium* species called distributive conjugal transfer (DCT), also considered as the fourth kind of HGT generating transconjugants from parental mosaic genomes [78].

## EVs AND HOST IMMUNE RESPONSE

A plethora of studies has highlighted the strength of EVs in inducing host humoral and cellular immune response. EVs have been shown to induce immune response with varying success in different gram-positive bacteria. Olaya *et al.* [57] showed that the vaccination of mice with EVs of *S. pneumoniae* elicits antibody production against virulent pneumococcal infections. Similarly, immunization of mice with *S. aureus* EVs showed protection from lethal challenge primarily due to Th1 cell-mediated and humoral immunity against *Staphylococcal* lung infections [79]. A vital role of cell-mediated protection is in clearing the invading pathogen from the host, and EVs are efficient in developing cell-mediated immunity [80]. EVs from *B. anthracis* are immunogenic in BALB/c mice in which immunised mice developed a robust IgM response against toxin components. Therefore, vesicle-immunized mice survive for a longer period than the controls after *B. anthracis* challenge [13].

In gram-positive bacteria, arrays of molecules involved in immune evasion have been demonstrated. For example, *S. aureus* EVs, which contain coagulase enzymes and factors, mediate clot formation when added into the serum [10, 81]. Thus, EVs might help in the formation of fibrin networks surrounding pathogens, forming an environment with limited access to the innate immune system. EVs from *S. aureus* also contain super-antigens, lipase, and protein A, which help bacteria to evade the immune system. EVs and associated proteins from lactobacilli modulate the activity of immune cells by affecting host innate and adaptive immune responses [82]. Since EVs are biologically active, they can cause disease without associating with the live cells. The *in vitro* and *in vivo* studies show that EVs from *S. aureus* upregulates pro-inflammatory mediators that elicit the Th17 response with increased production of IgE causing atopic dermatitis-like inflammation [11]. It also induced both Th1 and Th17 neutrophilic pulmonary inflammation facilitating airway hypersensitivity against inhaled allergens [12]. The pathogen-derived EV associated proteins modulate pro-inflammatory properties. It was observed that mice survived longer after *S. pneumoniae* challenge when PspA, a surface protein, packaged in the EVs of a gram-negative bacteria *Salmonella enterica* strain that was used for intranasal immunization [83]. In *Mycobacterium*, Gehring *et al.* [84] suggested that the EVs from pathogenic strains are enriched in lipoproteins, which are considered as TLR2 agonists. Again using *Mycobacterium* species and *Bacillus Calmette-Guerin* (BCG), Prados Rosales *et al.* [67] reported that the modulation in immune response via the cargo content of EVs is TLR2-dependent. A study examined the effect of immunization between EV and BCG in a mouse model to determine their vaccine potential. EV of *M. tuberculosis* showed higher efficacy against *M. tuberculosis* infection without any adjuvant when compared to BCG in mouse immunization [85].

## ROLE OF EVs IN BIOFILM FORMATION

Over the years, a striking feature of bacteria that has gained importance is their ability to form biofilms. Biofilm formation confers persistence under diverse environmental conditions, resistance to antimicrobial agents, and facilitates colonization in the host [70]. Bacterial vesicles contribute to biofilm formation and transfer DNA to other bacteria sharing genes involved in antibiotic resistance [86–88]. A plethora of studies on the role of EVs in biofilm formation has been investigated in *B. subtilis, M. ulcerans,* and *Streptococcus mutans* [34, 60, 89]. EVs were isolated from the *B. subtilis* biofilm [34]. Similar sized EVs have been isolated from both planktonic and biofilm cultures of gram-positive bacteria [34, 85]. Marsollier *et al.* [60] showed that *M. ulcerans* adopt a biofilm structure with an ECM containing vesicles that are highly cytotoxic. In *L. monocytogenes*, EVs were isolated from planktonic cultures but not from its biofilm [39]. It has been observed that extracellular DNA (eDNA) is one of the significant parts of the biofilm matrix that is sufficient to allow the adherence in the early stages of biofilm formation [90]. Liao *et al.* [89] demonstrated that *S. mutans* produce eDNA by multiple avenues, including lysis-independent MVs in the planktonic state. Thus, eDNA in EVs of planktonic cultures may play some role in biofilm production and bacterial colonization. Moreover, the vesicle-associated extracellular DNA contributes to biofilm formation by influencing the structural integrity and stability of the biofilm [89]. One of the strategies employed by bacteria to survive in high density is quorum sensing, which produces quorum sensing molecules called autoinducers that help in bacterial adherence and biofilm formation [91]. In *S. aureus*, the quorum-sensing related *agr* locus controls the expression of the cell wall surface proteins and virulence factors found on the pathogen and its EVs [10, 92]. The virulence factors have been identified within *S. aureus* EVs, suggesting a link between *agr* locus and production of EVs [93].

## EVs AS A VACCINE PLATFORM

EVs from pathogenic bacteria have the potential to be harnessed as vaccines. By using a lethal sepsis model, it was shown that the *Staphylococcal* EVs could be used as a potential vaccine. In this study, EVs from genetically engineered *S. aureus* mutant expressing detoxified cytolysins were found to be immunogenic in mice [18]. Previously, Choi *et al.* [79] showed the potential role of native *S. aureus* derived EVs in inducing adaptive immunity and T-cell responses with no toxicity in mice. They also suggested that the protection conferred by the EVs in immune response was without any adjuvants, thus indicating a powerful vaccine strategy. In contrast, OMV based vaccines against gram-negative 330 bacteria hamper the vaccine application due to the toxicity of lipopolysaccharide [18, 94]. In mycobacteria, EVs are reported to be immunogenic. Few studies showed that the immune response is directed against lipoproteins and bacterial cellular components present in EVs. These studies emphasized the importance of identification of EV-associated components and their role in generating protective immunity, thereby 334 incorporating artificial EVs as vaccine platforms [95].

EVs are stable in the biological fluids, preserve proteins from degradation, and act as an antigenic compound to fight against infections. They are considered the most superior substitute in vaccine delivery. The attention is growing more towards biological compounds than synthetic ones and is an ideal choice in therapeutics. In this context, further investigation on bio-engineered extracellular vesicles would improve the remaining complexities and skepticism.

## CONCLUSION AND PERSPECTIVE

EVs are meant for biological transmission from one cell to another. The secretory pathway fine-tunes the natural processes and regulates the host immune response via intercellular communication among bacteria, fungi, and mammalian cells. Gram-positive bacteria constitute a wide range of pathogenic species causing clinical manifestations. As reviewed here, the naturally occurring EVs play a critical role in intercellular communication between bacteria as well as between bacteria and host. This review describes how gram-positive EVs play a role in biological events and can be potential targets for vaccine development. Compared to OMVs derived from gram-negative bacteria, the biology of gram positive EVs remains undetermined, especially in terms of biogenesis, composition, and uptake. Phenotypic differences in their outer membrane composition and virulence-associated factors may need further investigation to interpret the exact process of vesiculogenesis and their role in the causation of diseases. EVs proteome analysis can pave the way for the identification of virulence factors, cytotoxic molecules, immune-associated components, and various other aspects associated with biogenesis, release, and application-based research meant for better input in vaccine platform and facilitation to humankind. To elucidate the mechanisms, our group focuses on the composition and genetic studies of EV biogenesis in *Vibrio cholera* and *S. aureus*. Besides, how the quorum-sensing system and its inhibitors regulate the composition of EVs and exchange of macro-molecules during biofilm formation will be informative. Since EVs are involved in HGT, they might play a crucial role in the dissemination of antibiotic resistance phenotype. Bacteria in biofilms are more resistant to antibiotics, and reports are suggesting that EVs from biofilm carried drug-binding proteins, therefore, showed their involvement in antibiotic resistance. Furthermore, it would be equally interesting to study the relationships of EVs with biofilm formation and biofilm matrix.

## Abbreviations:

BCG: – Bacillus Calmette-Guerin,
ECM: – extracellular matrix,
eDNA: – extracelllular DNA,
EV: – extracellular vesicle,
HGT: – horizontal gene transfer,
MGE: – mobile genetic element,
MV: – membrane vesicle,
OMV: – outer membrane vesicle,
PGN: – peptidoglycan,
PSM: – phenol-soluble modulin,
TB: – tuberculosis.
